# Unusual Symptomatic Multipartite Patella Associated with Quadriceps Fat Pad Edema

**DOI:** 10.5334/jbr-btr.945

**Published:** 2016-03-15

**Authors:** Zeynep Maras Ozdemir, Cemile Ayse Gormeli, Aysegul Sagir Kahraman, Gokhan Demirtas, Gokay Gormeli

**Affiliations:** 1 Inonu University School of Medicine, TR

**Keywords:** Multipartite, patella, symptomatic, quadriceps fat pad, edema

## Abstract

Partite patella is a known cause of anterior knee pain, especially in adolescents and young males, although most patients are asymptomatic. Symptomatic partite patella is usually revealed on magnetic resonance imaging (MRI) as bone marrow edema within the opposing bone fragments. We present a case of unusual bilateral symptomatic multipartite patella in an adult who presented with quadriceps fat pad edema and mass effect as well as slightly edematous signal changes within the bone fragments on MRI. This case indicates that symptomatic partite patella can also present with edema-like imaging findings in the adjacent soft tissue due to chronic mechanical irritation.

## Introduction

Bipartite or multipartite patella is a developmental variant resulting from the failure of the secondary ossification centers to fuse with the main body of the patella. Although most patients are asymptomatic, partite patella can also be one of the primary causes of knee pain [1]. Bone marrow edema within the bone fragments on knee magnetic resonance imaging (MRI) is usually the sole imaging finding for symptomatic bipartite patella [2].

The quadriceps (suprapatellar) fat pad (QFP) is an extrasynovial structure with a triangular shape and is one of the fat pads located in the anterior knee [3]. It fills the gap between the posterior part of the quadriceps tendon insertion and the retropatellar cartilage covering the proximal pole of the patellar base [3]. QFP edema or impingement syndrome, which may also be associated with anterior knee pain, is an inflammatory process within the quadriceps fat pad and may be analogous to Hoffa’s disease of the infrapatellar fat pad [4,5].

Here, we describe an unusual case of symptomatic multipartite patella, who presented with slightly edematous signal alterations within the bone fragments along with QFP edema with mass effect, both diagnosed on MRI. To the best of our knowledge, this is the first case of symptomatic partite patella associated with QFP edema to be described in the literature.

## Case Report

**Figure 1:**
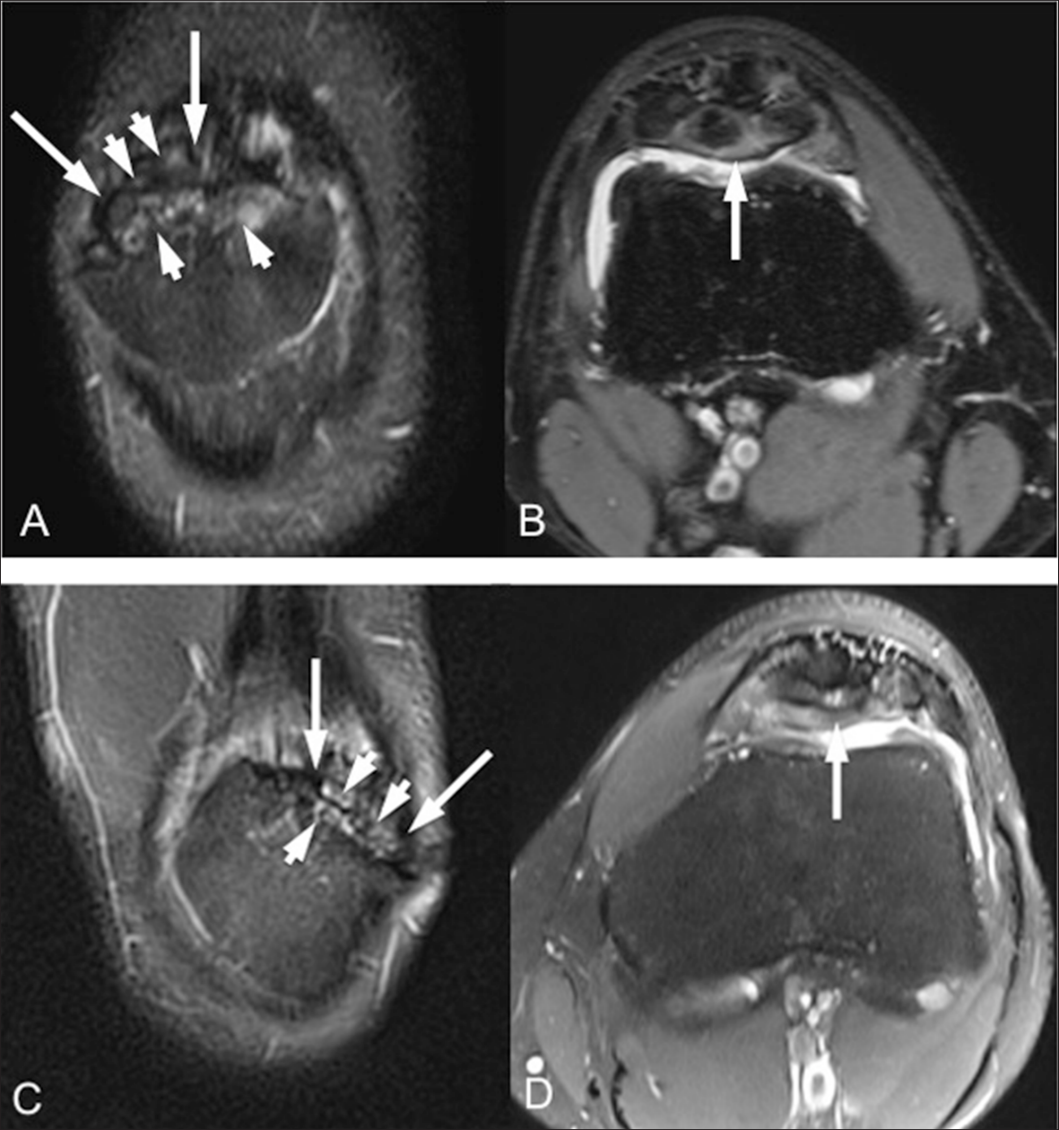
Coronal fat-saturated T2-weighted (A, C) and axial fat-saturated proton-density-weighted (B, D) MR images (respectively for right and left knee) show bilateral multipartite patella variation (long arrows) with bone marrow edema-like signal changes within the bony fragments (short arrows).

**Figure 2:**
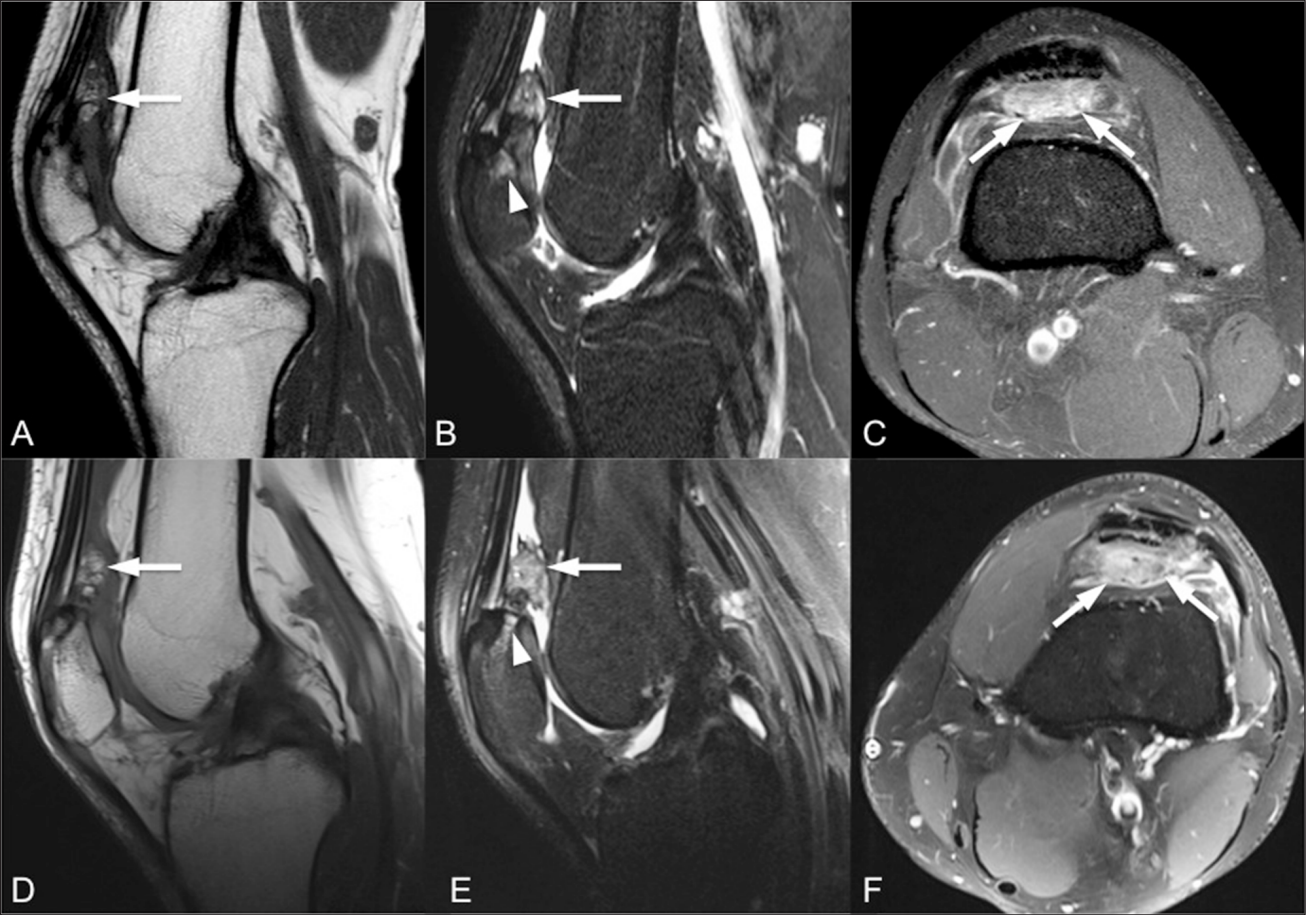
Sagittal T1-weighted (A, D), sagittal fat-saturated T2-weighted (B, E) and intravenous contrast-enhanced axial fat-saturated T1-weighted (C, F) MR images (top row for right knee, bottom row for left knee) show enlarged and inflamed quadriceps fat pad indicated by increased signal intensity with convex posterior border and contrast enhancement by intravenous contrast administration (arrows).

A 29-year-old male, who is a construction worker, presented with a six-month history of bilateral anterior knee pain and underwent an MRI examination at our department. He had no history of major knee trauma. The intensity of his pain was moderate to severe at rest and increased when seated or in a squatting position. On physical examination, there was point tenderness over the suprapatellar region on both sides. No clinical or laboratory findings that supported the presence of inflammation or infection were identified. A standard anteroposterior radiograph and MRI examinations revealed bilateral multipartite patella variation. The MRI also revealed slight bone marrow edema within the main patellar fragment and nonfused bony fragments (Figure 1). In addition, the MRI showed QFP edema and inflammation that was characterized by increased signal of the fat pad along with mass effect on the suprapatellar joint recess and contrast enhancement by intravenous contrast administration (Figure 2). Surgical treatment was proposed for removing the unstable bony fragments, but the patient refused and received conservative treatment, including oral nonsteroidal anti-inflammatory drugs (NSAIDs) and physical therapy. There was pain reduction at the two- and six-month follow-ups compared to baseline; however, he reported transient pain and discomfort after strenuous activities at the same follow-ups.

## Discussion

Bipartite/multipartite patella is a developmental variation which is commonly detected incidentally. Although most patients are asymptomatic, partite patella can also be one of the primary causes of anterior knee pain [1,6] (Table 1). The bipartite patella was categorized by Saupe into three subgroups based on the location of the fragments in 1921: Type I involving the inferior pole, Type II the lateral margin, and Type III the superolateral quadrant of the patella [1,7,8]. There is a fibrocartilaginous tissue between the partite fragment and the main patella [1,8,9]. This tissue is reported as fibrous tissue and fibrocartilage, fibrocartilage only, and fibrocartilage and hyaline cartilage by various authors [1].

**Table 1:** Common Causes of Anterior Knee Pain.

	Traumatic Disorders	Nontraumatic Disorders

Patellar disorders	Patellar fractures, patellar dislocation, post-traumatic osteochondral lesions	Patellofemoral osteoarthritis, chondromalacia/articular cartilage lesions, tumours, infection, osteochondritis dissecans, multipartite-bipartite patella
Quadriceps/patellar tendon disorders	Quadriceps tendon tear, patellar tendon tear	Quadriceps tendinosis, patellar tendinosis (jumper’s knee), Osgood-Schlatter, Sinding-Larsen-Johansson disease
Supra- and infrapatellar fat pad disorders	–	Hoffa’s disease, excessive patellar tendon-lateral femoral condyle friction syndrome (superolateral Hoffa’s fat pad edema), quadriceps fat pad edema inflammation, tumours
Bursae, plica, and recesses disorders	–	Bursitis, synovial plica syndrome, tumours

Two basic mechanisms have been accepted to explain the reasons for triggering a symptomatic status: single, direct trauma, and repetitive minor injuries [12]. Acute or overuse injuries may cause disruption of the synchondrosis or pseudarthrosis. This disruption allows the bone fragments to move abnormally. Excessive motion may then lead to friction, impaction between the bone fragments, and, eventually, bone edema [1,2]. On the other hand, an excessive traction force on the partite fragment by the lateral retinaculum and vastus lateralis with repeated flexion and extension generates abnormal stress on the synchondrosis or pseudarthrosis [1,9]. Moreover, a recent study reported the presence of patellofemoral malalignment, medialized and laterally tilted patella compared to the contralateral intact side, in symptomatic bipartite patella [10].

The QFP is the smallest of the three normal fat pads located in the anterior knee and is bordered anteriorly by the distal quadriceps tendon, inferiorly by the patella with retropatellar cartilage surface, and posteriorly by the suprapatellar recess of the knee joint [3]. QFP edema or impingement syndrome is an inflammatory process within the quadriceps fat pad and may be analogous to Hoffa’s disease of the infrapatellar fat pad [4,5]. Anatomical abnormalities of the extensor mechanism, including patellofemoral malalignment, have been described in the etiology of the patellar tendon-lateral femoral condyle friction syndrome in previous reports, whereas the pathophysiology of the quadriceps fat pad abnormalities remain poorly understood [11,12]. Roth et al. theorized that excessive knee flexion at high angles may cause QFP edema because of the repetitive microtrauma or development of an overuse injury [5]. To the best of our knowledge, neither patellar tendon-lateral femoral condyle friction syndrome nor other impingement syndromes of the knee by partite patella has previously been described. It probably depends on location of the partite fragments which are most commonly found superolaterally [1].

Here, we have described that QFP edema with mass effect as well as slightly edematous signal changes within the bone fragments on MRI in a patient with symptomatic partite patella. However, Kavanagh et al. reported that bone marrow edema within the partite fragments was the sole imaging finding in almost 50 percent of their patients with symptomatic partite patella [2]. In our case, the bilateral QFP edema related to the partite patella supports the hypothesis that mechanical impingement of bone onto soft tissue is a cause. Partite fragments adjacent to the QFP may cause friction or impingement between the bone and soft tissue and lead to the development of edema of the QFP due to repeated knee flexion and extension during daily routine or sports activities.

In conclusion, QFP edema with mass effect on MRI may be a reason or contributing factor for the explanation of complaints in patients with symptomatic partite patella. Therefore, it should be assessed in patients with partite patella and persistent anterior knee pain.

## Competing Interests

The authors declare that they have no competing interests.
